# In vitro assessment of the effect of probiotic lactobacillus reuteri on peri-implantitis microflora

**DOI:** 10.1186/s12903-021-01762-2

**Published:** 2021-08-19

**Authors:** Munaz Mulla, Mushir Mulla, Shashikanth Hegde, Ajit V. Koshy

**Affiliations:** 1 Department of Periodontology, Yenepoya Dental College, University road, Deralakatte, Mangalore, Karnataka 575018 India; 2 Department of Oral and Dental Health, College of Applied Health Sciences in Arrass, Qassim University, Buraydah, Kingdom of Saudi Arabia; 3 Department of Oral Pathology and Microbiology, M.A. Rangoonwala College of Dental Sciences and Research Centre, Pune, Maharashtra India

**Keywords:** Peri-implantitis, Probiotics, Lactobacillus reuteri, Minimum inhibitory concentration (MIC)

## Abstract

**Background:**

Probiotics affect both the development and stability of microbiota by altering the colonization of pathogens and thus helps in stimulating the immune system of the individual. The aim of the present study is to assess the effect of probiotics on peri-implantitis microflora, by determining the minimum inhibitory concentration (MIC) of *Lactobacillus reuteri,* that can be effectively administered as an antimicrobial agent on specific peri-implantitis pathogens. Hence, this study will be helpful in finding the MIC of *L. Reuteri* that can be effectively administered as an antimicrobial agent on specific peri-implantitis pathogens.

**Methods:**

This experimental research was conducted on patients visiting the periodontology department in M. A. Rangoonwala college of dental sciences and research centre. Sub-gingival plaque samples were collected from peri-implantitis patients to identify various peri-implantitis microorganisms. The identified microorganisms were compared to each other and Chi-Square test was used to calculate statistical significance. The isolated microorganisms were subjected to the effect of probiotic *Lactobacillus reuteri* in-vitro*.* Minimum inhibitory concentration (MIC) was assessed using serial dilution method.

**Results:**

The research results showed the presence of *Porphyromonas gingivalis*, *Aggregatibacter actinomycetemcomitans, Prevotella intermedia, Streptococcus salivaris* and *Staphylococcus aureus* in the subgingival samples from peri-implantitis patients. Statistically, significantly higher proportion of samples had *Porphyromonas gingivalis*. When subjected to the effect of *L. reuteri,* all the microorganisms were affected by *L.reuteri* except *Aggregatibacter actinomycetemcomitans.*

**Conclusion:**

This study provides the various MIC value for each isolated pathogen against *L.reuteri.* The authors recommend to avoid using standard guidelines for probiotic dose in the treatment of peri-implant infections as the antimicrobial profile is different for each periodontal pathogen.

## Background

Several treatment options are available to replace missing teeth. But over the years, placing dental implants are considered as the best treatment modality [1]. Just like any natural tooth, gum diseases can also affect the dental implants leading to peri-implantitis.

Peri-implantitis is an inflammatory process, which has an effect on the tissues surrounding an osseo-integrated implant, leading to the loss of supporting bone [2]. Every affected implant is always a threat to the durability of the associated prosthetic replacement. Peri-implant diseases are common finding following implant therapy as peri-implant mucositis is seen among 30.7% of all dental implants, and peri-implantitis is seen among 9.6% of all the dental implants [3–6]. Evaluation of the literature has shown that the microbiota related to peri-implantitis is more complex when compared to a healthy peri-implant conditions. The microbiota consists mostly of anaerobic gram-negative bacteria [7]. Even in dental implants, a cause-effect relationship has been identified between the accumulation of bacterial plaque and the development of inflammatory changes in the soft tissues surrounding oral implants. Thus, procedures are now aimed to prevent and treat any such peri-implantitis through different methods. These include mechanical debridement, surgical therapy with or without regenerative procedures, local or systemic antibiotics.

Various new strategies are also introduced to manage this infection such as by recreating the healthy microbial environment by the use of non-pathogenic microorganisms. This helps to stimulate the host immunity by inhibiting the growth of pathogenic microorganisms. One of the strategies is the use of Probiotics to manage peri-implantitis. Probiotics are live microorganisms that are administered in sufficient amounts to produce a beneficial effect on the host animal [8]. Minimum inhibitory concentration (MIC) is the lowest concentration of a drug that inhibits the visible growth of test organism. In vitro detection of MIC of a drug against pathogens will act as a guideline for its in vivo application. MIC scores are usually used to identify an effective dose of the drug against the pathogen [9].

The aim of the present study is to assess the effect of probiotics on peri-implantitis microflora, by determining the minimum inhibitory concentration (MIC) of *Lactobacillus reuteri.* Thus, this study will be helpful in finding the MIC of *L. Reuteri* that can be effectively administered as an antimicrobial agent on specific peri-implantitis pathogens.

## Methods

Patients with peri-implantitis were recruited from the periodontology department of M.A.Rangoonwala college of dental sciences and research Centre, Pune from June, 2016 to July, 2019. The participants for the research were selected based on the selection criteria. The inclusion criteria were: Age-18 years and above, patients presenting at least one implant with peri-implantitis (an implant with a probing depth of ≥ 4 mm), signs of peri-implantitis (loss of supporting bone as estimated on radiographs, bleeding on probing or suppuration), no implant mobility. The exclusion criteria were: Consumption of any form of tobacco, taking medications for any systemic disease, presence of systemic diseases, use of any other oral hygiene aid other than toothbrush and dentifrice, undergone periodontal therapy in the last 6 months, undergone peri-implantitis treatment during the last 6 months. Written informed consents were obtained from all participants based on the guidelines of the Declaration of Helsinki. This study was approved by the Institutional Ethics Committee of M.A.Rangoonwala college of dental sciences and research Centre, Pune.

To avoid the inter-examiner bias, only one principal investigator was employed to collect the samples. Thirty-five patient samples were collected based on the selection criteria. Microbial samples were collected from the peri-implantitis pockets at the following visit. Prior to sampling, the supra-gingival plaque was removed with a sterile curette. Cotton rolls were used to isolate the site along with air syringe to gently dry the area. Subgingival samples were obtained by inserting #30 sterilized paper points into the deepest probing point and kept for 30 s. The paper points were then removed and immediately placed in a sterile Eppendorf tube prepared with transportation medium of 2 ml of Thioglycollate broth (0.4% agar, 0.15% Thioglycollate buffered saline). Immediately after the collection of samples, they were transferred in an ice box to the microbiological lab. Each sample was made 2 sets, one of which was kept to incubate under aerobic condition and the other under anaerobic condition.

The sample was mixed thoroughly and 5 µl aliquots were inoculated using a sterile loop onto petri plates of Blood Agar & MacConkey’s Agar for the Aerobes and Kanamycin Blood Agar & Bacteroides Bile Esculin (BBE) Agar for Anaerobes.

For Aerobic, plates were kept in an incubator for 24 h at 37 °C. For Anaerobic, plates were kept in Gas pack anaerobic jar for 5–6 days at 37 °C. After incubation multiple distinguished colonies were observed on the plates, which were further sub cultured individually to obtain pure colonies of each. The pure colonies were then identified using colony, morphological and biochemical characteristics as per the standard Bergey’s Manual of Systematic Bacteriology. Chi-Square test was used to calculate statistical significance between the identified organisms.

The periodontal organism which were isolated was then subjected to the effect of probiotic Lactobacillus reuteri in-vitro. *L.reuteri* was cultured in Rogosa Agar-(Selective medium), incubated at 37 °C for 3–4 days.

Serial tube dilution technique was followed in this study to detect the MIC due to its ability to determine antimicrobial activity along with its MIC and is based on the guidelines of the Clinical and Laboratory Standards Institute [9]. Following are the steps for the MIC procedure used in this study:

1. 9 dilutions of lactobacilli strain were done with Thioglycollate broth for MIC.
2. In the initial tube 20 µl of lactobacilli strain was added into the 380 µl of Thioglycollate broth.
3. For dilutions 200 µl of Thioglycollate broth was added into the next 9 tubes separately.
4. Then from the initial tube 200 µl was transferred to the first tube containing 200 µl of Thioglycollate broth. This was considered as 10^−1^ dilution.
5. From 10^−1^ diluted tube 200 µl was transferred to second tube to make 10^−2^ dilution.
6. The serial dilution was repeated up to 10^−9^ dilution.
7. From the maintained stock cultures of peri-implantitis organisms, 5 µl was taken and added into 2 ml of Thioglycollate broth.
8. In each serially diluted tube 200 µl of above culture suspension was added.
9. The tubes were incubated for 48–72 h in anaerobic jar at 37 °C and observed for turbidity, which indicates the growth of the organisms

Media Composition of Thioglycollate medium with hemin and vit K (For 1L) as follows: Tryptose-15gms, Yeast extract-10gms, Sodium thioglycollate-0.50gms, Sodium chloride-2.5gms, L-cystine HCL-0.5gms. The least concentration of *L.Reuteri* in the tube, which does not show any turbidity, was considered as MIC of *L.Reuteri* for that particular test pathogen.

## Results

Thirty-five samples were collected from 35 peri-implantitis pockets in 35 patients. Out of them 21 were females and 14 males, aged from 46 to 73 years with a mean of 53 years. Thirty implants were located in the posterior sextant and 5 implants in the anterior sextant. Twenty-three implants were placed in the mandible and the others in the maxilla.

Each patient sample was subjected to microbial testing to identify the peri-implantitis microorganisms. Five types of microorganisms were detected in 35 samples: *Porphyromonas gingivalis (P.gingivalis)*, *Aggregatibacter actinomycetemcomitans (A.a), Prevotella intermedia(P. intermedia), Streptococcus salivaris (Strep.salivaris), Staphylococcus aureus (Staph.aureus)* (Table 1). Significantly higher proportion of samples had *P.gingivalis* isolated (*P*-value < 0.001). Distribution of type of organism isolated differs significantly in the study sample (*P*-value < 0.001). Chi-Square test was used to calculate statistical significance. Each identified microorganism from the different samples was cultured separately. It was subjected to the effect of probiotic *L.Reuteri*. MIC value was calculated for each microorganism against L.Reuteri (Table 2). MIC value: *P.gingivalis*—25 mg/mL, *P.intermedia*—100 mg/mL*, Strep.salivaris*—1.6 mg/mL*, Staph.aureus*—6.25 mg/mL. *A.a* was unaffected by *L. reuteri*.

**Table 1:** Distribution of number of samples the particular organism was identified

Sr. no	Organism name	No. of samples	% of samples	*P*-value
1	*Porphyromonas gingivalis*	20	57.1	0.001***
2	*Aggregatibacter actinomycetemcomitans*	6	17.1	
3	*Prevotella intermedia*	10	28.6	
4	*Streptococcus salivaris*	15	42.9	
5	*Staphylococcus aureus*	10	28.6	

*P*-value by One-sample Chi-Square test. ***P-value < 0.001 statistically significant for unequal distribution

**Table 2:** Effect of probiotic *lactobacillus reuteri* concentrations on different microorganisms

Probiotic concentrations	100 mg/ml	50 mg/ml	25 mg/ml	12.5 mg/ml	6.25 mg/ml	3.12 mg/ml	1.6 mg/ml	0.8 mg/ml	0.4 mg/ml	0.2 mg/ml
*Pg*										
1	S	S	S	R	R	R	R	R	R	R
2	S	R	R	R	R	R	R	R	R	R
3	S	R	R	R	R	R	R	R	R	R
4	R	R	R	R	R	R	R	R	R	R
5	R	R	R	R	R	R	R	R	R	R
6	R	R	R	R	R	R	R	R	R	R
7	R	R	R	R	R	R	R	R	R	R
8	S	R	R	R	R	R	R	R	R	R
9	R	R	R	R	R	R	R	R	R	R
10	R	R	R	R	R	R	R	R	R	R
11	S	S	S	R	R	R	R	R	R	R
12	S	R	R	R	R	R	R	R	R	R
13	S	R	R	R	R	R	R	R	R	R
14	S	R	R	R	R	R	R	R	R	R
15	R	R	R	R	R	R	R	R	R	R
16	R	R	R	R	R	R	R	R	R	R
17	S	S	S	R	R	R	R	R	R	R
18	S	R	R	R	R	R	R	R	R	R
19	S	R	R	R	R	R	R	R	R	R
20	S	R	R	R	R	R	R	R	R	R
*Aa*										
1	R	R	R	R	R	R	R	R	R	R
2	R	R	R	R	R	R	R	R	R	R
3	R	R	R	R	R	R	R	R	R	R
4	R	R	R	R	R	R	R	R	R	R
5	R	R	R	R	R	R	R	R	R	R
6	R	R	R	R	R	R	R	R	R	R
*Pi*										
1	R	R	R	R	R	R	R	R	R	R
2	R	R	R	R	R	R	R	R	R	R
3	S	R	R	R	R	R	R	R	R	R
4	R	R	R	R	R	R	R	R	R	R
5	R	R	R	R	R	R	R	R	R	R
6	R	R	R	R	R	R	R	R	R	R
7	S	R	R	R	R	R	R	R	R	R
8	R	R	R	R	R	R	R	R	R	R
9	R	R	R	R	R	R	R	R	R	R
10	R	R	R	R	R	R	R	R	R	R
*Strep. Salivaris*										
1	R	R	R	R	R	R	R	R	R	R
2	R	R	R	R	R	R	R	R	R	R
3	R	R	R	R	R	R	R	R	R	R
4	R	R	R	R	R	R	R	R	R	R
5	R	R	R	R	R	R	R	R	R	R
6	S	S	S	S	S	S	S	R	R	R
7	S	S	S	S	S	S	S	R	R	R
8	S	S	S	S	S	S	S	R	R	R
9	R	R	R	R	R	R	R	R	R	R
10	S	S	S	S	S	S	S	R	R	R
11	S	S	S	S	S	S	S	R	R	R
12	S	S	S	S	S	S	S	R	R	R
13	R	R	R	R	R	R	R	R	R	R
14	R	R	R	R	R	R	R	R	R	R
15	S	S	S	S	S	S	S	R	R	R
*Stap. Aureus*										
1	R	R	R	R	R	R	R	R	R	R
2	S	S	S	S	S	R	R	R	R	R
3	R	R	R	R	R	R	R	R	R	R
4	S	S	S	S	S	R	R	R	R	R
5	R	R	R	R	R	R	R	R	R	R
6	S	S	S	S	S	R	R	R	R	R
7	R	R	R	R	R	R	R	R	R	R
8	S	S	S	S	S	R	R	R	R	R
9	R	R	R	R	R	R	R	R	R	R
10	S	S	S	S	S	R	R	R	R	R

S – Sensitive; R- Resistant*; Pg-Porphyromonas gingivalis*; *Aa-Aggregatibacter actinomycetemcomitans*; *Pi- Prevotella intermedia*; *Strep.Salivaris—Streptococcus salivaris*; *Stap. Aureus -Staphylococcus aureus*

## Discussion

The present study was conducted to assess the effect of Probiotic *L. Reuteri* on the identified micro-organisms from Peri-implantitis patients. Microorganisms tend to play an important role in peri-implantitis [10]. Peri-implantitis has been described as a site specific inflammatory bacterial condition [11]. Presence of plaque biofilm around implants is considered as primary cause for peri-implantitis. The presence of periodontopathic bacteria is generally considered to be a risk factor for periimplantitis [12]. Many studies have reported the high prevalence of bacteria such as *P.gingivalis* and *A.a* [13–18]*.* Just like in periodontitis cases, treatment of peri-implantitis also aims at controlling the pathogenic bacterial infection. Some of the treatment options are scaling and systematic antibacterial therapy. Systemic antibacterial therapy has drawback of causing bacterial resistance in the subgingival flora [19–22].

A probiotic that could alter the oral microbial ecology may be a useful tool in the clinical management as it offers two-fold benefits [23]. Firstly, it reduces the immunogenicity of the oral microbiota by inhibiting the periodontal pathogens. Secondly, it leads to immune homeostasis and reduction of the inflammation by modulating the active disease-associated immune/inflammatory pathways.

*L.reuteri* is considered as a probiotic organism. *L reuteri*, is a gram-positive bacterium belonging to the genus *Lactobacillus* [24]. *L. reuteri* improves clinical parameters, crevicular fluid volume and cytokine levels by decreasing the IL-1ß and IL-8 levels in patients with peri-implant mucositis [25]. *Lactobacillus* has been shown to prevent the growth of *A.a* (88%), *P.gingivalis* (82%), and *P.intermedia* (65%) in patients with periodontal disease [26, 27].

The present study aimed to assess the effect of probiotics on peri-implantitis microflora. Thus, the peri-implantitis microflora was assessed to identify the presence of different microorganisms. All the subgingival samples were subjected to microbiological examination and tested for the presence of eight different types of microorganisms.

Anaerobes: *A.a, P.gingivalis, P.intermedia, Fusobacterium nucleatum, Staphylococcus aureus, Tannerella forsythia, Bacterioides forsythus.* Aerobes: *Strep.salivaris.* These microorganisms were selected based on their frequent presence in peri-implantitis microflora [17, 18]. Five types of microorganisms were detected in 35 samples in this study: *P.gingivalis, A.a, P.intermedia, Strep.salivaris*, *Staph.aureus.* Higher proportion of samples had *P.gingivalis*.

Persson GR and Renvert S., 2014 conducted a study to find bacterial species that were at higher counts from implants with peri-implantitis and found: *A.a, Campylobacter rectus, Helicobacter pylori, Haemophilus influenzae, P.gingivalis, Staph.aureus, Streptococcus intermedius, Streptococcus mitis, Tannerella forsythia, Treponema denticola* [28]*.* It has also been reported that patients with peri-implantitis shows the presence of Staph. aureus and enteric rods [7, 29]. The microflora of periimplantitis when compared to periodontitis showed higher counts of *A.a, P.intermedia, P.gingivalis, Treponema denticola*, and *Tannerella forsythia* [30, 31]. Present study showed significantly higher proportion of *P.gingivalis* isolated from the samples. Reports suggest that oral infection with *P. gingivalis* induces bone loss in subjects with implants [9, 32, 33].

Minimum inhibitory concentration (MIC) is a significant diagnostic tool to identify the resistance of microorganisms to any antimicrobial agent. It can also help to monitor the activity and potency of new antimicrobial agents [34]. Different bacterial species have different MICs. Sensitive strains show relatively low MICs and resistant strains shows relatively high MICs. In this study, the five isolated peri-implantitis microorganisms were subjected to the effect of Probiotic *L. reuteri*. This was done to identify the different MIC values of *L. reuteri* for the five identified micro-organisms. Apart from *A.a,* all other microorganism were susceptible to *L.reuteri.* Whereas in other studies *A.a* strains were found to be the most susceptible species to *lactobacilli* [9]. To determine the exact value of MIC for *A.a,* further dilutions are required. Based on this study, it can be stated that probiotic *L.reuteri* concentration of 100 mg/ml can be used against *P.gingivalis, P.intermedia, Strep.salivaris*, *Staph.aureus,* in the treatment of periimplantitis.

The effect of *L. reuteri* on periodontopathogenic bacteria have been studied in the past but the effect on peri-implanitis microflora is scarce. Various reports have been documented stating that the probiotic supplementation when used as an adjunct to scaling and root planning are having clinical and microbiological effect in the treatment of chronic periodontitis [9, 35–40]. *L.reuteri* has also been considered as an adjuvant therapy for treatment of peri-implantitis as oral care probiotics [25, 38, 41, 42] Whereas some authors found no adjunctive effects [43]. Not many literatures have been published on the use of probiotics for treatment of peri-implantitis. Thus, no conclusive result can be withdrawn and more research needs to be carried out to determine the effect of use of probiotics in the treatment of peri-implantitis. The present in vitro MIC study will help us to design and conduct future clinical trial and thus detect the beneficial effect of *L.reuteri* on patients at risk for peri-implantitis. Apart from clinical and microbiological factors, future research should also focus on inflammatory markers such as IL-1β, IL-6 and IL-8 to identify the benefit of use of probiotics in the treatment of peri-implantitis. More knowledge on the healing mechanism will also help to improve the treatment options.

Patients with Periodontitis and Peri-implantitis present higher levels of inflammatory markers such as IL-6, TGF-1β, IL-1β, and IL-8 compared to healthy patients [44–46]. Peri-implanitis can also have an impact on the systemic status through these inflammatory markers [47, 48]. Thus further research can be done on the said inflammatory markers, to identify the benefit of use of probiotics in the treatment of peri-implantitis.

## Conclusion

The present in vitro MIC study will help us to design and conduct future clinical trial and thus detect the beneficial effect of *L.reuteri* on patients at risk for peri-implantitis. This study provides the various MIC value for each isolated pathogen against *L.reuteri*. As the MIC values differs between the pathogen, it would be recommended that standard guidelines for probiotic dose in the treatment of peri-implant infections should not be recommended, as major differences in the antimicrobial profile of major periodontal pathogens were found.

## Data Availability

The datasets used and/or analysed during the current study are available from the corresponding author on reasonable request.

## Abbreviations

MIC: Minimum inhibitory concentration
*L.Reuteri*: *Lactobacillus reuteri*
*P.gingivalis*: *Porphyromonas gingivalis*
*A.a*: *Aggregatibacter actinomycetemcomitans*
*P. intermedia*: *Prevotella intermedia*
*Strep.salivaris*: *Streptococcus salivaris*
*Staph.aureus*: *Staphylococcus aureus*
